# Magnitude of Drug–Drug Interactions in Special Populations

**DOI:** 10.3390/pharmaceutics14040789

**Published:** 2022-04-04

**Authors:** Sara Bettonte, Mattia Berton, Catia Marzolini

**Affiliations:** 1 Division of Infectious Diseases and Hospital Epidemiology, Departments of Medicine and Clinical Research, University Hospital Basel, 4031 Basel, Switzerland; mattia.berton@unibas.ch (M.B.); catia.marzolini@usb.ch (C.M.); 2 Faculty of Medicine, University of Basel, 4031 Basel, Switzerland; 3 Department of Molecular and Clinical Pharmacology, University of Liverpool, Liverpool L69 3GF, UK

**Keywords:** drug interaction, special populations, elderly, obese, pregnant women

## Abstract

Drug–drug interactions (DDIs) are one of the most frequent causes of adverse drug reactions or loss of treatment efficacy. The risk of DDIs increases with polypharmacy and is therefore of particular concern in individuals likely to present comorbidities (i.e., elderly or obese individuals). These special populations, and the population of pregnant women, are characterized by physiological changes that can impact drug pharmacokinetics and consequently the magnitude of DDIs. This review compiles existing DDI studies in elderly, obese, and pregnant populations that include a control group without the condition of interest. The impact of physiological changes on the magnitude of DDIs was then analyzed by comparing the exposure of a medication in presence and absence of an interacting drug for the special population relative to the control population. Aging does not alter the magnitude of DDIs as the related physiological changes impact the victim and perpetrator drugs to a similar extent, regardless of their elimination pathway. Conversely, the magnitude of DDIs can be changed in obese individuals or pregnant women, as these conditions impact drugs to different extents depending on their metabolic pathway.

## 1. Introduction

Drug–drug interactions (DDIs) occur when a drug, called a “perpetrator”, modifies the pharmacokinetic or pharmacodynamic effects of a second drug, called a “victim”, leading to either toxicity or treatment failure. Pharmacokinetic DDIs can occur at the level of drug absorption, distribution, metabolism, and renal excretion. The main mechanisms include drug chelation, gastric pH- or mobility-dependent changes in absorption, protein binding displacement, and the inhibition or induction of drug metabolizing enzymes and drug transporters [1]. In some cases, DDIs are deliberate and aim to improve the exposure of the victim drug, such as the use of ritonavir to boost protease inhibitors of HIV (e.g., darunavir), COVID-19 (i.e., nirmatrelvir), and HCV (i.e., paritaprevir). The risk of DDIs increases with the number of co-administered medications and is therefore expected to be higher in elderly or obese individuals as they are more likely to present with comorbidities. These special populations, in addition to the population of pregnant women, are characterized by physiological changes that can modify drug pharmacokinetics and, consequently, the magnitude of DDIs and their related management. To date, this clinically relevant question has not been addressed thoroughly.

This review summarizes the physiological changes in elderly, obese, and women in the third trimester of pregnancy, as well as the impact of these changes on drug absorption, distribution, metabolism, and excretion. Furthermore, it provides a compilation of DDIs studies in elderly, obese, or pregnant populations for which a control group without the condition of interest was included. The impact of physiological changes on the magnitude of DDIs was then analyzed by comparing the exposure of a medication in the presence and absence of an interacting drug for the special population relative to the comparator population.

## 2. Materials and Methods

### 2.1. Data Sources

Literature searches of the PubMed and Embase databases were conducted to identify all relevant DDI studies in special populations up to February 2022. The search was performed by combining keywords and/or MeSH (Medical Subject Headings) terms referring to DDIs and the population of interest. Keyword and MeSH terms used for elderly were ‘age’, ‘aged’, ‘aging’, ‘elderly’, or ‘young’ plus ‘drug interactions’. For the obese populations, the search was performed using ‘obese’, ‘obesity’, ‘BMI’ (body mass index), ‘overweight’, and ‘drug interactions’. Lastly, for the pregnant women population, the keywords and MeSH terms used were ‘pregnant women’, ‘pregnancy’, ‘postpartum’, plus ‘drug interaction’. The results of each search were imported and managed in EndNote V. 20.2. In addition to the results obtained from the literature searches, reference lists of the selected studies were screened to identify additional references.

### 2.2. Inclusion Criteria

As inclusion criteria, the definitions of “elderly” and “obese” were considered. An “elderly” individual is defined as a person aged 60 years old or more, as per the definition of the World Health Organization (WHO) [2]. Furthermore, according to WHO, an “obese” individual is defined as having a body mass index (BMI) equal or greater than 30 kg/m^2^ [3]. Pharmacokinetic studies are generally performed in the third trimester of pregnancy (i.e., 29–40 weeks from last menstrual cycle); thus, the pregnant women included in this review were in their third trimester. The corresponding comparator populations consisted of healthy individuals aged between 20 to 55 years old, with a BMI between 18.5 and 25 kg/m^2^ and non-pregnant or after delivery (i.e., mostly 4–5 weeks post-partum). From the literature search, in vitro data, non-human in vivo data, and results from physiologically based pharmacokinetic (PBPK) modelling were not considered. The remaining results were screened, first by the title, then by the abstract, and finally by full-text evaluation. The studies were included if they met the following criteria: (i) DDIs in the standard population and special population were investigated in the same study; (ii) primary pharmacokinetic parameters were reported; (iii) the individuals in the study were mostly of white ethnicity except for the pregnant population, which also included studies conducted in other ethnicities due to the limited number of clinical studies in general with pregnant women. The pharmacokinetic data measured during pregnancy and in non-pregnant women or postpartum were compared within the same ethnicity. DDI studies with metabolic inhibitors were only available for ritonavir or cobicistat in combination with the HIV protease inhibitor or elvitegravir. Since these antiretroviral agents cannot be given unboosted, the magnitude of DDIs for inhibition was evaluated by comparing the pharmacokinetic parameters of the boosted antiretroviral agent during the third trimester and postpartum.

### 2.3. Data Extraction

The data relevant for the current study were extracted from the results section or tables and collated in an Excel file for further evaluation and analysis. If the data of interest were only reported as figures, GetData Graph Digitizer^®^ V. 2.26 was used to extract numerical values and in the case of pharmacokinetic profiles, a non-compartmental analysis was conducted to derive the primary PK parameters. As for the paper by Hodel et al., 2019 [4], the standard deviation of the ratio (i.e., special population/comparator) was not calculated because the number of individuals participating in the study was <30 and therefore not sufficient to test the normality assumption, due to the heterogeneity between studies and due to the variation in the study measurements.

## 3. Results

For the elderly population, the search yielded 87 results and additional studies were found in the paper by Stader et al. [5]. Four studies were duplicates; the others were excluded since they did not match the inclusion criteria. Finally, 11 studies were included in this analysis. For obesity, the research yielded 168 results, of which 7 were duplicates. Finally, only three studies matched the inclusion criteria. For the pregnant population, 268 studies were found, of which 5 were duplicates. Based on the literature search and the reference list of Bukkems et al. [6], 29 studies fulfilled our inclusion criteria.

The subsequent sections summarize the physiological changes for each population of interest and their effect on the magnitude of DDIs is presented as the ratio of a medication exposure in presence and absence of an interacting drug for the population of interest relative to the corresponding control group. It should be noted that DDIs with a ratio outside the 0.8–1.25 interval (bioequivalence criterion) may be of potential clinical relevance. DDIs are organized according to the impacted drug process (i.e., absorption, metabolism, or renal elimination) and the effect of the DDI (i.e., inhibition or induction).

### 3.1. Elderly Population

#### 3.1.1. Physiological Changes

With advancing age, the body undergoes a series of anatomical, physiological, and biochemical modifications. Several studies have been conducted to investigate the effect of physiological changes in the elderly on drug pharmacokinetics. Contradictory results have been reported for the effect of age on the absorption process [7]. However, studies have consistently shown that drug distribution is changed in the elderly due to the higher proportion of adipose tissue, the lower total body water, and albumin levels [7]. Metabolism is decreased due to a reduction in liver weight and hepatic blood flow, whereas the abundance of cytochromes (CYPs), uridine diphosphate-glucuronosyltransferases (UGTs), and hepatic transporters was found not to be age dependent [8]. Drug excretion is reduced in the elderly due to the decrease in kidney weight and renal blood flow and, consequently, in glomerular filtration rate [8]. Finally, a reduction in cardiac output has also been reported [8]. Altogether, these changes translate to higher drug exposure; thus, caution is notably needed when prescribing drugs with a narrow therapeutic index (e.g., theophylline [9]) in the elderly.

#### 3.1.2. Magnitude of DDIs Impacting Drug Absorption

The magnitude of DDIs impacting drug absorption was evaluated only in one study including a group of elderly and young subjects [10]. The study assessed the co-administration of metoclopramide, an antiemetic drug known to increase gastric motility and gastric emptying time [11,12] in conjunction with the beta blocker metoprolol. The study found a 30% and 10% increase in metoprolol exposure and half-life, respectively, in elderly compared to young individuals, which can be explained by the physiological decline in the liver and kidney weights and blood flow with aging. However, when metoprolol was co-administered with metoclopramide, the resulting DDI magnitude was similar in elderly and young individuals, suggesting that aging, per se, does not significantly impact gastric mobility or emptying and, consequently, DDIs occurring via this mechanism (Table 1). Of note, metoclopramide inhibits CYP2D6 [13], the main enzyme metabolizing metoprolol [14]. Therefore, this study suggests that intestinal inhibition of CYP2D6 is not altered in the elderly compared with young individuals.

#### 3.1.3. Magnitude of DDIs Impacting Metabolism

##### Inhibition

The literature search identified five studies that determined the magnitude of DDIs with metabolic inhibitors in both elderly and young individuals [9,15,16,17,18]. The evaluated drugs were mainly metabolized by cytochrome P450 (CYP) 1A2 (i.e., antipyrine [19], theophylline [9]) and CYP3A enzymes (i.e., antipyrine [19], oxycodone [18]) (Table 2). The metabolic inhibitors included the H2-blocker cimetidine, a weak inhibitor of CYP1A2 and CYP3A4; the antibiotic ciprofloxacin, a strong inhibitor of CYP1A2; and the strong CYP3A4 inhibitor clarithromycin [20]. In all studies except Cohen et al. [16], the exposure and the half-life of drugs were 26% and 30% higher, respectively, in elderly individuals compared to young individuals, regardless of the presence of the perpetrator drug. As mentioned earlier, the higher drug exposure in the elderly is caused by the physiological decline of hepatic and renal mass and blood flow with aging. These physiological changes have been previously shown to impact drugs in a similar way, regardless of the drug pharmacokinetic characteristics [7]. This explains that the magnitude of DDIs with metabolic inhibitors is mostly similar in elderly and young adults. Furthermore, this observation supports previous work reporting that CYP1A2 and CYP3A4 activities are not impacted by aging [21,22,23,24].

##### Induction

The literature search yielded four studies comparing the induction effect in elderly and young adults [25,26,27,28]. The victim drugs evaluated in these studies were substrates of CYP1A2 and CYP3A4 (Table 3). The metabolic inducers included the anticonvulsant phenytoin, a moderate inducer of CYP1A2; the anti-tuberculosis drug rifampicin, a strong inducer of CYP3A [20]; and the sedative drug dichloralphenazone. Elderly individuals presented a 6% increase in drug exposure and a 15% longer half-life of both the victim (and the perpetrator drugs); hence, the DDI magnitude with metabolic inducers was mostly comparable with young adults (Table 3). Altogether, these data indicate that DDIs caused by either metabolic inducers or inhibitors are not dependent on age and therefore DDIs can be managed in the elderly in the same way as for young individuals.

#### 3.1.4. Magnitude of DDIs Impacting Renal Elimination

The magnitude of DDIs impacting renal secretion was evaluated for the dopamine agonist amantadine, a substrate of renal organic cation transporter (OCT) 2 [29]. This drug was co-administered with inhibitors of this renal transporter [30], namely the antimalarial drug quinine and the antiarrhythmic drug quinidine [31,32,33]. The amantadine area under the curve (AUC) was 38% higher in the elderly as a result of age-related physiological changes. However, given that aging impacts drugs in a similar way, the DDI ratio was found to be comparable in elderly and young individuals (Table 4). This observation implies also that aging does not significantly alter the activity of the renal transporter OCT2.

#### 3.1.5. Summary

Available studies indicate that aging does not significantly change the magnitude of DDIs. This observation is explained by the fact that the activity of drug metabolizing enzymes or transporters is not altered with aging. Importantly, aging impacts extent the exposure of the victim and perpetrator drugs to a similar extent and therefore the magnitude of the DDI remains unchanged compared to young individuals. Thus, DDIs in elderly can a priori be managed in the same way as for young individuals. However, DDIs comprising a narrow therapeutic index should be approached with caution considering that drug exposure is higher in elderly. It should be emphasized that the individuals enrolled in these studies did not present severe comorbidities; thus, one cannot exclude that the presence of severe comorbidities, such as renal impairment, could have an impact on the DDI magnitude [34].

### 3.2. Obese Population

#### 3.2.1. Physiological Changes

Obesity is characterized by physiological, hemodynamical, and biological changes that can modify drug disposition. Studies evaluating drug absorption in obese individuals are limited and their results are contradictory; some report higher absorption and others report unchanged absorption [35,36,37,38,39,40,41]. Several studies evaluated the effect of obesity on drug distribution. All studies agree that the larger adipose and muscle tissues in obese individuals function as reservoirs where the drugs (especially lipophilic drugs) can distribute to and then, during the elimination phase, be slowly released; therefore, obesity leads to a decrease in maximum concentration (C_max_) and an increase in half-life [36,42]. Drug metabolism is influenced by various physiological parameters such as the liver weight, the splanchnic blood flow, the amount of hepatocytes or microsomal protein per gram liver, and the enzyme abundance. From the literature, it is clear that both liver weight and splanchnic blood flow are higher in obese individuals. CYP3A4 activity (in vitro data) and abundance have been shown to be lower; however, less is known about hepatocytes or microsomal protein per gram of liver or for other enzyme abundances [43,44,45]. From the clinical data, it seems that clearance and drug exposure are dependent on the metabolic pathway of a given drug. CYP3A4 abundance has been shown to be lower in obese individuals; conversely, liver blood flow is greater. These opposite changes seem to compensate each other since the clearance of CYP3A4 substrates was shown to be lower or unchanged [46]. On the other hand, UGT substrates have a higher clearance, resulting in lower AUC and trough concentration at steady state (C_trough_) in obese individuals [46]. Renal clearance is strictly linked to glomerular filtration rate and the latter has been reported to be significantly higher in obese individuals, thereby explaining the higher drug elimination observed in clinical trials [47].

#### 3.2.2. Magnitude of DDIs Impacting Metabolism

##### Inhibition

Only two DDI studies comparing the impact of inhibition in lean and obese individuals were found in the literature [48,49]. Both studies used the antifungal posaconazole (UGT1A4 substrate [50]) and a strong CYP3A4 inhibitor [20], while the victim drugs were the anti-anginal drug ranolazine (CYP3A > CYP2D6 substrate [51]) in one case and the antipsychotic drug lurasidone (CYP3A4 substrate [52]) in the other. Obesity-related physiological changes resulted in a 32% reduction in posaconazole exposure in obese individuals compared to lean individuals due to the higher clearance of UGT substrates, while the opposite was found for the washout half-life, due to the higher distribution in adipose tissue. On the other hand, the AUCs of CYP3A substrates were comparable in obese individuals and lean individuals (ranolazine 6955 ng·h/mL versus 6454 ng·h/mL; lurasidone 60.6 ng·h/mL versus 63.1 ng·h/mL), while the half-life was higher in obese individuals (ranolazine 6.02 versus 4.99 h; lurasidone 10.9 versus 9.4 h). In both studies, the DDI magnitude was found to be lower in obese individuals compared to lean individuals for the AUC and C_max_ ratios, except for the ranolazine C_max_ ratio, which was similar between the two groups (Table 5). Notably, the authors found that the AUC of the victim drug was proportional to the concentration of posaconazole, with less pronounced DDI magnitudes when posaconazole exposure was lower.

##### Induction

The literature search identified only one study comparing the inducing effect in lean and obese individuals [53]. The anticonvulsant drug topiramate (mainly eliminated unchanged renally [54]), an inducer, was co-administered with the oral contraceptive ethinylestradiol (sulfation, UGT, and CYP3A4 substrate [53]). At a dose of 200 mg, the inducing effect of topiramate was weak [55] and no difference for the DDI magnitude was observed between groups (Table 6).

#### 3.2.3. Summary

Available DDI studies with metabolic inhibitors seem to indicate that obesity-related physiological changes could differently impact the magnitude of DDIs depending on the metabolic pathway of the perpetrator drug. For instance, the exposure of inhibitors undergoing UGT metabolism or highly lipophilic drugs is expected to be lower in obese individuals due to their changed physiology. A decreased exposure of the inhibitor may consequently result in less inhibition and therefore a lower DDI magnitude. For induction, only one study was found and suggested a similar DDI magnitude in obese and lean individuals, albeit with the weak inducer topiramate. Based on data with metabolic inhibitors, one could hypothesize that moderate or strong inducers whose exposure may be reduced in obese individuals could lead to a lower DDI magnitude given that the inducing effect has been demonstrated to be dose dependent [55,56,57,58,59]. The current literature search revealed important knowledge gaps, as no DDI studies in obese individuals with moderate or strong inducers were found. Furthermore, there is a limited number of studies with inhibitors so, altogether, there is a need for more DDI studies in obese individuals to confirm our assumptions.

### 3.3. Pregnant Women

#### 3.3.1. Physiological Changes

From the beginning of the gestation period to the end of pregnancy, several physiological changes occur. These modifications can impact drug pharmacokinetics and potentially impair drug efficacy. Notably, an increase in progesterone levels leads to a decrease in intestinal mobility and can therefore modify drug absorption [60,61]. Pregnancy results in major hemodynamic changes such as plasma volume expansion leading to a larger volume of distribution of drugs (e.g., hydrophilic drugs) [60]. Furthermore, the distribution can be impacted by the gradual decrease in albumin level due to its dilution into the large plasma volume as well as the slight reduction in α1-acidic glycoprotein (AAG) levels affecting the distribution of highly bound drugs [60,62]. In addition, the drug distribution is affected by the progressive increase in fat mass weight and in the absolute fat mass blood flow [62]. The hepatic metabolism is not affected by the increase in liver weight, as confirmed by Dallmann et al. [62], but the higher rate of metabolism observed in pregnant women is a result of CYP induction by progesterone and estrogen [60]. The higher rate of metabolism is also explained by an increase in cardiac output during pregnancy, which is related to an increase in heart weight [62]. Finally, the kidney volume, the effective renal plasma flow, and the glomerular filtration rate increase during pregnancy, leading to higher clearance of renally eliminated drugs [60,62].

#### 3.3.2. Magnitude of DDIs Impacting Drug Absorption

DDIs impacting absorption during pregnancy were found for the antiretroviral drugs tenofovir diproxil fumarate (TDF) in combination with ritonavir and for tenofovir alafenamide (TAF) given with either ritonavir or cobicistat [63,64,65]. TDF and TAF are prodrugs that are converted to tenofovir after absorption. Unlike tenofovir, the prodrugs are substrates of the transporters P-glycoprotein (P-gp) and breast cancer resistance protein (BCRP) [66]. Thus, the DDI with ritonavir and cobicistat occurs at the intestinal level, whereby inhibition of these efflux transporters by ritonavir or cobicistat leads to increased absorption of TDF or TAF. The DDI magnitude was comparable between pregnant and non-pregnant women (Table 7). This suggests that the intestinal boosting effect of ritonavir or cobicistat is not modified during pregnancy. However, the pregnancy-related physiological changes decrease the systemic tenofovir (active moiety) C_trough_ and AUC by approximately 30%, regardless of the prodrug considered.

#### 3.3.3. Magnitude of DDIs Impacting Metabolism

##### Inhibition

DDI studies with metabolic inhibitors were only available for ritonavir or cobicistat in combination with HIV protease inhibitors or elvitegravir. As these antiretroviral agents are not used unboosted, the magnitude of DDIs for inhibition was evaluated by comparing the pharmacokinetic parameters of the boosted antiretroviral agents during the third trimester and postpartum. The exposures of atazanavir, darunavir, and elvitegravir boosted with cobicistat were shown to be significantly reduced during the third trimester of pregnancy compared to postpartum. On average, AUC, C_max_, C_trough_, and half-life were 40%, 30%, 80%, and 55% lower, respectively. This effect relates to the hormonal changes during pregnancy causing CYP3A4 induction, which, combined with other physiological changes, results in lower exposures of these antiretroviral agents notably when boosted with cobicistat. As indicated by the studies listed in Table 8, the C_trough_ of antiretrovirals boosted with cobicistat was much lower (reduction by 70–90%) compared to ritonavir boosting (10–50%). This difference is explained by the fact that cobicistat concentrations during the dosing interval drop below the half maximal inhibitory concentration for CYP3A4 inhibition in pregnant woman, thereby impairing the boosting effect [6]. Conversely, ritonavir concentrations in pregnant women do still exceed the half maximal inhibitory concentrations for CYP3A4 inhibition after twice daily dosing [6]. Furthermore, ritonavir has been shown to be a more robust pharmacokinetic booster in presence of inducers even in non-pregnant individuals [67].

##### Induction

The literature search yielded three DDI scenarios conducted in African women using the moderate inducer efavirenz [20] together with the antimalarials lumefantrine (CYP3A4 substrate [86]), artemether (CYP3A4/CYP2B6 substrate [86]), and piperaquine (CYP3A4/CYP2C8 substrate [87]). The studies reported in Table 9 show that the AUC ratios in presence and absence of efavirenz are lower in pregnant women compared to non-pregnant individuals for all three substrates [88,89,90,91,92,93,94]. It has been suggested that the inducing effect of pregnancy on CYP enzymes, especially CYP2B6 (the main enzyme metabolizing efavirenz [20]), may lead to a lower efavirenz exposure and therefore a lower induction potential [90]. This could be explained by the dose-dependent induction effect, together with the lower plasma concentration of the inducer as seen for carbamazepine and phenytoin [95,96]. However, more studies are needed to confirm this hypothesis either with strong inducers or with efavirenz considering that the study participants were not genotyped for CYP2B6. Thus, one cannot exclude that a higher proportion of CYP2B6 slow metabolizers in the control group leads to higher efavirenz concentrations and related induction [97].

#### 3.3.4. Summary

The magnitude of DDIs and the related clinical relevance may change during pregnancy depending on the site where the DDI occurs. Pregnancy did not significantly impact the extent of DDIs in which inhibitors are used to enhance drug absorption via the inhibition of intestinal drug transporters. However, the magnitude of the DDIs was shown to differ if the systemic exposure of the perpetrator drug was reduced due to pregnancy-related physiological changes. The decrease in the concentrations of an inhibitor is expected to mitigate the magnitude of a DDI. In the case of a pharmacokinetic booster this would lead to a reduced boosting effect, thereby potentially leading to suboptimal exposure of the boosted drug. Suboptimal boosting of cobicistat during pregnancy led to the contraindication of its use during pregnancy [98]. The decrease in the concentrations of an inducer due to the physiological changes in pregnancy would also be expected to mitigate the magnitude of a DDI given the dose-dependent effect of induction. More studies are needed to confirm this assumption.

## 4. Discussion

Physiological changes in aging, obesity, and pregnancy were shown to have different impacts on DDIs. Elderly individuals are distinct from obese individuals and pregnant women since both the victim and perpetrator drugs are impacted in a similar way; therefore, the magnitude of DDIs occurring at the level of the liver or kidney is unchanged. Conversely, obesity and pregnancy impact the magnitude of DDIs as these conditions affect drugs to different extent depending on their metabolic pathway. In obese individuals, the magnitude of DDIs involving perpetrators metabolized by CYP3A4 could remain unchanged whereas those involving perpetrators undergoing UGT metabolism could be reduced. Additionally, one could extrapolate that the magnitude of DDIs occurring via the inhibition of renal transporters could also be mitigated if the exposure of the perpetrator drug is reduced. Pregnancy-related physiological changes generally lead to a reduced exposure of the perpetrator drug which, in turn, contributes to mitigation of DDIs happening at the hepatic or kidney levels. This effect can be problematic when DDIs are deliberate, such as for the pharmacokinetic boosters ritonavir and cobicistat. Available studies suggest that physiological changes have a minimal impact on DDIs occurring at the intestinal level, likely explained by the high drug concentrations in the intestine, which saturate the metabolic or transport activities and thereby prevail over any physiological effect. Further studies are needed to confirm this assumption, notably in obese individuals.

For some populations (i.e., obese individuals and pregnant women), the variations in the level of plasma proteins could have an impact on the magnitude of DDIs, although this has not been evaluated thoroughly. Thus, future research is also needed to determine whether changes in protein binding impact the magnitude of DDIs and require dose adjustment. The studies reported in this review evaluated DDIs mostly for drugs administered intravenously or orally. The intramuscular long-acting administration of drugs has attracted much interest as a means to improve treatment adherence. Long-acting drug administration was initially used for the treatment of schizophrenia and for contraception [99], and is now increasingly considered for the treatment and prevention of HIV infection, malaria, and tuberculosis. For instance, the first long-acting intramuscular antiretroviral drugs for treatment and/or prevention of HIV infection (cabotegravir and rilpivirine, i.e., Cabenuva^®^ and Apretude^®^) were approved in 2020 and 2021, respectively. To date, several long-acting drugs are in development for HIV treatment and prevention (i.e., lenacapavir, islatravir). The long-acting drugs are slowly released from the injection site into the systemic circulation thereby allowing infrequent administration (i.e., monthly or bimonthly), which may improve long-term treatment adherence [99]. The intramuscular administration of drugs presents the disadvantage that, once injected, the drug release cannot be interrupted; this may be problematic in the case of new occurring disease requiring a treatment with an inducer or inhibitor of drug metabolism. There is currently a lack of data on DDIs with long-acting intramuscular drugs which complicates their management in clinical practice. Thus, further studies are warranted to evaluate the magnitude of DDIs with the long-acting intramuscular administration of drugs both in standard and special populations.

## 5. Conclusions

This review provides a comprehensive analysis of the physiological changes in elderly, obese, and pregnant populations and their impact on the magnitude of DDIs. Information provided in this review could be used to design DDI clinical studies in special populations and help with the interpretation of the results. Sufficient research has been conducted in elderly individuals to conclude that aging does not impact significantly the magnitude of DDIs. For obese individuals and pregnant women, the data were sparse and further studies are needed. However, it is clear that the magnitude of DDIs depend on physiological changes, such as enzyme abundance or hepatic blood flow, and the metabolic pathways of the victim and perpetrator drugs.

## Figures and Tables

**Table 1:** Comparison of DDI magnitudes impacting absorption in elderly individuals relative to young individuals.

			Ratio Presence/Absence Perpetrator		
Victim Drug	Perpetrator Drug	Study Subjects (Age, Sex)	AUC	Half-Life	Reference
Metoprolol	Metoclopramide	mean age 33 (3 ♂, 5 ♀)	1.07	1.03	[10]
100 mg (PO), single dose	10 mg (IV), single dose	mean age 81 (6 ♂, 1 ♀)	1.06	0.91	

Legend: AUC, area under the curve; IV, intravenous administration, PO, oral administration. The arithmetic mean of the raw pharmacokinetic data was used to calculate the corresponding ratios.

**Table 2:** Comparison of DDI magnitudes with inhibitors impacting drug metabolism in elderly individuals relative to young individuals.

			Ratio Presence/Absence Perpetrator		
Victim Drug	Perpetrator Drug	Study Subjects (Age, Sex)	AUC	Half-Life	Reference
Antipyrine	Cimetidine	mean age 24 (6 ♂)	1.40	-	[15]
8 mg/kg (PO), single dose	200 mg (PO), QID, single dose	mean age 72 (6 ♂)	1.38	-	
CYP1A2/CYP3A					
Theophylline	Cimetidine	mean age 28 ± 5 (9 NS)	1.29	1.37	[16]
5 mg/kg (PO), single dose	200 mg (PO), QID, steady state	mean age 67 ± 4 (9 NS)	1.40	1.45	
CYP1A2					
Theophylline	Cimetidine	mean age 28 ± 5 (9 NS)	1.45	1.59	[16]
5 mg/kg (PO), single dose	300 mg (PO), QID, steady state	mean age 67 ± 4 (9 NS)	1.58	1.72	
CYP1A2					
Theophylline	Cimetidine	mean age 27 ± 1 (10 ♂)	1.41	1.38	[17]
10 mg (IV), single dose	400 mg (PO), TID, steady state	mean age 76 ± 2 (10 ♂)	1.40	1.32	
CYP1A2					
Theophylline	Cimetidine	mean age 25 ± 2 (8 ♂)	1.31	1.41	[9]
5 mg/kg (IV), single dose	400 mg (PO), BID, steady state	mean age 28 ± 1 (8 ♀)	1.42	1.43	
CYP1A2		mean age 71 ± 1 (8 ♂)	1.36	1.31	
		mean age 72 ± 2 (8 ♀)	1.33	1.36	
Theophylline	Ciprofloxacin	mean age 25 ± 2 (8 ♂)	1.49	1.51	[9]
5 mg/kg (IV), single dose	500 mg (PO), BID, steady state	mean age 28 ± 1 (8 ♀)	1.50	1.48	
CYP1A2		mean age 71 ± 1 (8 ♂)	1.42	1.40	
		mean age 72 ± 2 (8 ♀)	1.40	1.45	
Theophylline	Cimetidine + ciprofloxacin	mean age 25 ± 2 (8 ♂)	1.64	1.73	[9]
5 mg/kg (IV), single dose	CIM: 400 mg (PO), BID, steady state	mean age 28 ± 1 (8 ♀)	1.79	1.75	
CYP1A2	CIP: 500 mg (PO), BID, steady state	mean age 71 ± 1 (8 ♂)	1.64	1.64	
		mean age 72 ± 2 (8 ♀)	1.60	1.68	
Oxycodone	Clarithromycin	mean age 22 (6 ♂, 4 ♀)	1.84	1.32	[18]
10 mg (PO), single dose	500 mg (PO), BID, steady state	mean age 74 (7 ♂, 3 ♀)	2.09	1.19	
CYP3A					

Legend: AUC, area under the curve; BID, twice a day; CIM, cimetidine; CIP, ciprofloxacin, IV, intravenous administration; NS, not specified; PO, oral administration; QID, four times a day; TID, three times a day. The arithmetic mean of the raw pharmacokinetic data was used to calculate the corresponding ratios.

**Table 3:** Comparison of DDI magnitudes with inducers impacting drug metabolism in elderly individuals relative to young individuals.

			Ratio Presence/Absence Perpetrator		
Victim Drug	Perpetrator Drug	Study Subjects (Age, Sex)	AUC	Half-Life	Reference
Antipyrine	Dichloralphenazone	mean age 29 (5 ♂, 3 ♀)	0.76	0.68	[25]
18 mg/kg (PO), QD, single dose	20 mg/kg (PO), QD, steady state	mean age 77 (3 ♂, 3 ♀)	0.97	0.87	
CYP1A2/CYP3A4					
Theophylline	Phenytoin	mean age 25 ± 1 (10 ♂)	0.63	0.72	[26]
5.6 mg/kg (IV), single dose	30 or 100 mg (PO), BID, steady state	mean age 73 ± 2 (10 ♂)	0.69	0.70	
CYP1A2					
S-hexobarbitone	Rifampicin	mean age 29 (6 NS)	0.16	0.41	[27]
500 mg (PO), single dose	600 mg (PO), QD, steady state	mean age 71 (6 NS)	0.17	0.43	
CYP unknown					
R-hexobarbitone	Rifampicin	mean age 29 (6 NS)	0.01	0.71	[27]
500 mg (PO), single dose	600 mg (PO), QD, steady state	mean age 71 (6 NS)	0.05	0.87	
CYP unknown					
Midazolam	Rifampicin	mean age 27 ± 4 (14 ♂)	0.08	0.48	[28]
3–8 mg (PO), single dose	600 mg (PO), QD, steady state	mean age 26 ± 4 (14 ♀)	0.11	0.41	
CYP3A4		mean age 70 ± 4 (10 ♂)	0.11	0.60	
		mean age 72 ± 5 (14 ♀)	0.11	0.33	
Midazolam	Rifampicin	mean age 27 ± 4 (14 ♂)	0.51	0.50	[28]
0.05 mg/kg (IV), single dose	600 mg (PO), QD, steady state	mean age 26 ± 4 (14 ♀)	0.38	0.43	
CYP3A4		mean age 70 ± 4 (10 ♂)	0.48	0.58	
		mean age 72 ± 5 (14 ♀)	0.44	0.48	

Legend: AUC, area under the curve; BID, twice a day; IV, intravenous administration; NS, not specified; PO, oral administration; QD, once a day. The arithmetic mean of the raw pharmacokinetic data was used to calculate the corresponding ratios.

**Table 4:** Comparison of DDI magnitudes impacting renal elimination in elderly individuals relative to young individuals.

			Ratio Presence/Absence Perpetrator		
Victim Drug	Perpetrator Drug	Study Subjects (Age, Sex)	AUC	Half-Life	Reference
Amantadine	Quinine	mean age 33 (5 ♂, 4 ♀)	1.45	-	[30]
3 mg/kg (PO), single dose	200 mg (PO), single dose	mean age 66 (4 ♂, 5 ♀)	1.13	-	
OCT2					
Amantadine	Quinidine	mean age 33 (5 ♂, 4 ♀)	1.24	-	[30]
3 mg/kg (PO), single dose	200 mg (PO), single dose	mean age 66 (4 ♂, 5 ♀)	1.22	-	
OCT2					

Legend: AUC, area under the curve; OCT2, organic cation transporter 2; PO, oral administration. The arithmetic mean of the raw pharmacokinetic data was used to calculate the corresponding ratios.

**Table 5:** Comparison of DDI magnitudes with inhibitors impacting drug metabolism in obese individuals relative to lean individuals.

			Ratio Presence/Absence Perpetrator		
Victim Drug	Perpetrator Drug	Study Subjects (BMI, Sex)	AUC	C_max_	Reference
Ranolazine	Posaconazole	BMI 23.5 kg/m^2^ (7 ♂, 7 ♀)	3.88	2.16	[48]
500 mg (PO), single dose	300 mg (PO), QD, steady state	BMI 40.9 kg/m^2^ (5 ♂, 9 ♀)	2.80	2.18	
CYP3A4					
Lurasidone	Posaconazole	BMI 23.1 kg/m^2^ (6 ♂, 5 ♀)	5.75	4.00	[49]
20 mg (PO), single dose	300 mg (PO), QD, steady state	BMI 49.3 kg/m^2^ (6 ♂, 7 ♀)	4.34	2.91	
CYP3A4					

Legend: AUC, area under the curve; BMI, body mass index; C_max_, peak concentration; PO, oral administration; QD, once a day. The geometric mean of the raw pharmacokinetic data was used to calculate the corresponding ratios.

**Table 6:** Comparison of DDI magnitudes with inducers impacting drug metabolism in obese individuals relative to lean individuals.

			Ratio Presence/Absence Perpetrator		
Victim Drug	Perpetrator Drug	Study Subjects (BMI, Sex)	AUC	C_max_	Reference
Ethinylestradiol	Topiramate	BMI 22.8 kg/m^2^ (12 ♀)	0.97	0.95	[53]
0.035 mg (PO), steady state	200 mg (PO), steady state	BMI 32.5 kg/m^2^ (12 ♀)	0.97	0.94	
Sulfation, glucuronidation, CYP3A4					

Legend: AUC, area under the curve; BMI, body mass index; C_max_, peak concentration; PO, oral administration. The geometric mean of the raw pharmacokinetic data was used to calculate the corresponding ratios.

**Table 7:** Comparison of DDI magnitudes impacting absorption in pregnant women relative to non-pregnant individuals.

			Ratio Presence/Absence Perpetrator		
Victim Drug	Perpetrator Drug	Study Subjects (Gestational Age, Sex)	AUC	C_trough_	References
TDF	Ritonavir	PP (58 ♀)	1.28	1.13	[63]
300 mg (PO), QD, steady state	100 mg (PO), BID, steady state	3T (53 ♀)	1.30	1.26	
P-gp, BCRP					
TAF	Ritonavir/cobicistat	PP (25 ♀)	1.87	-	[64,65]
25 mg (PO), QD, steady state	100 mg/150 mg (PO), QD, steady state	3T (27 ♀)	1.58	-	
P-gp, BCRP					

Legend: 3T, third trimester; AUC, area under the curve; BCRP, breast cancer resistance protein; BID, twice a day; C_trough_, trough concentration at steady state; P-gp, P-glycoprotein; PO, oral administration; PP, postpartum; QD, once a day; TAF, tenofovir alafenamide; TDF, tenofovir diproxil fumarate. The geometric mean and the median of the raw pharmacokinetic data were used to calculate the corresponding ratios.

**Table 8:** Comparison of DDI magnitudes with inhibitors impacting drug metabolism in pregnant women relative to non-pregnant individuals.

			Ratio Third Trimester/Post-Partum				
Victim Drug	Perpetrator Drug	Study Subjects (Ethnicity)	AUC	C_max_	C_trough_	Half-Life	References
Atazanavir 300 mg (PO), QD, steady state	Ritonavir 100 mg (PO), QD, steady state	58% white, 42% black	0.66	0.65	0.65	1.00	[68,69]
Atazanavir 300 mg (PO), QD, steady state	Ritonavir 100 mg (PO), QD, steady state	78% black, 20% white, 2% others	0.70	0.75	0.71	0.59	[70,71,72,73]
Atazanavir 300 mg (PO), QD, steady state	Cobicistat 150 mg (PO), QD, steady state	55% black, 18% Hispanic, 9% white	0.46	0.53	0.32	0.47	[74]
Darunavir 600 mg (PO), BID, steady state	Ritonavir 100 mg (PO), BID, steady state	66% black, 33% white	0.78	0.76	0.89	1.12	[75]
Darunavir 800 mg (PO), QD, steady state	Ritonavir 100 mg (PO), QD, steady state	61% black, 39% white	0.68	0.76	0.50	0.59	[75,76,77,78]
Darunavir 800 mg (PO), QD, steady state	Cobicistat 150 mg (PO), QD, steady state	72% black, 14% white, 14% Hispanic	0.50	0.63	0.11	-	[79]
Lopinavir 400 mg (PO), BID, steady state	Ritonavir 100 mg (PO), BID, steady state	64% black, 23% Hispanic, 13% white	0.71	0.74	0.61	-	[80,81,82,83]
Elvitegravir 150 mg (PO), QD, steady state	Cobicistat 150 mg (PO), QD, steady state	68% black, 16% white, 16% Hispanic	0.60	0.74	0.15	0.44	[84,85]

Legend: AUC, area under the curve; BID, twice a day; C_max_, maximum concentration; C_trough_, trough concentration at steady state; PO, oral administration; QD, once a day. For all the drugs listed, the mechanisms of the DDI are via CYP3A4 and P-gp. The current value represents the weighted mean of the ratios found in the different studies.

**Table 9:** Comparison of DDI magnitudes with inducers impacting drug metabolism in pregnant women relative to non-pregnant individuals.

			Ratio Presence/Absence Perpetrator		
Victim Drug	Perpetrator Drug	Study Subjects (Gestational Age, Sex)	AUC	C_trough_	References
Lumefantrine	Efavirenz	NP ALONE: (12♂, 63 ♀) + EFV: (11 ♂, 53 ♀)	0.42	-	[88,89,90,91]
480 mg (PO), BID, steady state	600 mg (PO), QD, steady state	2T/3T ALONE: 2T 60%, 3T 40%, (26 ♀) + EFV: 2T 20%, 3T 80%, (35 ♀)	0.61	-	[88,89,90]
CYP3A4					
Piperaquine	Efavirenz	NP ALONE: (5 ♂, 5 ♀) + EFV: (3 ♂, 13 ♀)	0.57	0.74	[92]
960 mg (PO), x for 3 days	600 mg (PO), QD, steady state	3T ALONE: (31 ♀) + EFV: (27 ♀)	0.62	0.50	[93]
CYP3A4/CYP2C8					
Artemether	Efavirenz	NP ALONE: (12 ♂, 46 ♀) + EFV: (11 ♂, 19 ♀)	0.20	-	[91]
80 mg (PO), BID, steady state	600 mg (PO), QD, steady state	3T ALONE: (30 ♀) + EFV: (9 ♀)	0.55	-	[94]
CYP3A4/CYP2B6					

Legend: 2T, second trimester; 3T, third trimester; AUC, area under the curve; BID, twice a day; C_trough_ trough concentration at steady state; EFV, efavirenz; NP, non-pregnant; PO, oral administration; QD, once a day. The mean and the geometric mean of the raw pharmacokinetic data were used to calculate the corresponding ratios.

## Data Availability

The raw data that support the findings of this study are available upon reasonable request to the corresponding author.
